# A Prediction Model for 9‐Year Depression Risk Among Patients With COPD in China

**DOI:** 10.1155/da/5536644

**Published:** 2026-09-18

**Authors:** Cui Wang, Mengya Wang, Yanying Bi, Nan Zhang

**Affiliations:** ^1^ Zhejiang Chinese Medical University School of Nursing, Hangzhou, Zhejiang Province, China; ^2^ Weihai Second Hospital Affiliated to Qingdao University, Weihai, Shandong Province, China; ^3^ Xinjiang Medical University School of Nursing, Urumqi, Xinjiang Uygur Autonomous Region, China

**Keywords:** COPD, depression risk, prediction model

## Abstract

**Objectives:**

To establish a prediction model for the 9‐year risk of depression in individuals with chronic obstructive pulmonary disease (COPD) using survey data from a random, nationally representative population of Chinese adults with COPD.

**Methods:**

Data from 851 middle‐aged and older adults with COPD participating in the China Health and Retirement Longitudinal Study (CHARLS) were analyzed. Depression risk was described as the development of depression during follow‐up among participants who were free of depression risk at baseline (CHARLS 2011) and met the depression risk criteria at the 9‐year follow‐up (CHARLS 2020). Relationships between candidate predictive factors and depression risk were examined using logistic regression, with bootstrapping applied to validate the final model. Model discrimination was evaluated using the area under the curve (AUC) of the receiver operating characteristic (ROC) curve, while calibration was examined via the Hosmer‐Lemeshow test.

**Results:**

Of the 851 participants included in the analysis, 434 developed depression risk during the subsequent 9 years. The final multivariable model incorporated age, educational level, marital status, exercise levels, peak expiratory flow (PEF), and smoking. The discriminative ability of the model was good, with an AUC of 0.717 (95% CI: 0.683–0.751). The Hosmer‐Lemeshow test indicated satisfactory calibration (*χ* ^2^ = 9.400, *p* = 0.310). A simple clinical scoring system was also developed to facilitate quantitative estimation of depression risk.

**Conclusion:**

This predictive model may facilitate earlier identification of COPD patients likely to develop depression within 9 years and may help inform targeted preventive interventions.

## 1. Introduction

Chronic obstructive pulmonary disease (COPD) is relatively common during midlife and older age. It is characterized by airflow restrictions and progressively impaired pulmonary function and contributes markedly to worldwide morbidity and mortality [1]. In 2019, ~212.3 million people worldwide were affected by COPD, resulting in 3.3 million deaths and representing the third‐highest cause of death [2]. In China, ~100 million adults have COPD, and the disease accounts for ~945,000 deaths [3, 4]. COPD also imposes a considerable economic burden worldwide, with estimated losses reaching $4.326 trillion, of which China bears the largest share, at $1.36 trillion dollars [5]. Consequently, effective prevention and management of COPD represent important public health priorities.

In recent years, depression risk among patients with COPD has received increasing attention because the coexistence of these conditions can complicate disease management and rehabilitation. The reported prevalence of depression risk in patients with COPD ranges from 44.4% to 51% [6, 7], and 22.8% of patients who were free of depression risk at baseline developed depression risk during 4 years of follow‐up [8]. Depressive symptoms can substantially compromise treatment response and prognosis in individuals with COPD, with patients experiencing depression demonstrating poorer treatment compliance and less favorable therapeutic outcomes [9]. Depression has also been found to increase mortality risk by ~1.27 times in individuals with COPD [10] and induce a substantial additional economic burden [11]. Because COPD currently has no cure, effective management of the depression risk is particularly important for improving patients’ overall health and quality of life.

Despite the increased likelihood of individuals with COPD developing depression and its substantial clinical consequences, rates of diagnosis and treatment remain low, with fewer than one‐third of affected individuals receiving standardized care [12]. Given the adverse effects of comorbid depression on health outcomes and healthcare costs, reliable tools for predicting depression risk would assist in identifying individuals at risk, thereby enabling earlier intervention. However, most existing studies have relied on cross‐sectional data and have therefore provided limited information regarding the long‐term development of depression risk. A scientifically validated longitudinal prediction model could support earlier recognition of individuals at elevated risk and facilitate timely intervention.

### 1.1. Aim

This study sought to establish a model for predicting the 9‐year likelihood of depression development in patients with COPD using survey data derived from a randomized, nationwide population of Chinese adults with COPD.

## 2. Methods

### 2.1. Participants

Data were obtained from the China Health and Retirement Longitudinal Study (CHARLS), which was a longitudinal survey of a population aged 45 years and above considered to be nationally representative. The CHARLS design involves four stages of stratified cluster probability sampling of 450 village units over 150 counties and 28 provinces [13]. The study baseline was in 2011, with subsequent 2‐yearly surveys involving standardized biomedical instruments and structured questionnaires [13]. In‐person interviews were conducted by trained research assistants to collect information on demographics, health‐related behaviors, and anthropometric measurements [13]. Participants were considered to have COPD if they reported a physician diagnosis of the disease. The present study used five waves of CHARLS data collected between 2011 and 2020. Participants were excluded if they (1) lacked COPD, (2) were <45 years of age, (3) had missing depression risk data at the 9‐year follow‐up, or (4) had more than 50% missing data for key study variables. A total of 1781 individuals aged ≥45 years with COPD completed the baseline assessment of depression risk. Among these individuals, 851 had no depression risk at baseline and had complete follow‐up data available for the 9‐year observation period; these participants constituted the final analytical cohort.

### 2.2. Ethical Approval

All participants gave written informed consent. The CHARLS protocol was approved by the Institutional Review Board of Peking University (approval number: IRB00001052‐11015). Study procedures were conducted as per the relevant guidelines and regulations.

### 2.3. Outcome

The primary outcome was the risk of incident depression within 9 years of follow‐up. Depression risk was evaluated using the 10‐item short form of the Center for Epidemiological Studies Depression Scale (CES‐D). Each item was rated using a 4‐point Likert scale, with 0 representing rarely or never and 3 indicating most or all of the time, yielding an overall score between 0 and 30. Items reflecting positive affect, including “feeling hopeful about the future” and “feeling happy,” were reverse‐scored. A total CES‐D score of ≥12 was considered indicative of depression risk.

## 3. Predictor Variables

### 3.1. Demographic Characteristics

The demographic variables examined in this study were age, sex, residence (urban or rural), marital status, and education. Marital status was classified as married/cohabiting versus single/divorced/widowed/separated. Educational attainment was classified into three levels: high school or above, middle school, and elementary school or below.

### 3.2. Health‐Related Behavior

Health‐related behavioral variables were history of smoking (current/former/never), alcohol intake (current/former/never), and exercise levels. Exercise levels were categorized by intensity, as described in the International Physical Activity Questionnaire Short Version [14]. Vigorous‐intensity activities were assigned a value of 8.0 metabolic equivalents (METs) and included heavy lifting, farming, digging, aerobic exercise, and fast cycling. Moderate‐intensity activities were assigned 4.0 METs and included light lifting, regular cycling, mopping, Tai Chi, and brisk walking. Low‐intensity activities were assigned 3.3 METs and included routine walking at home or work and leisure walking. Weekly physical activity was quantified by multiplying the MET value assigned to each activity by its duration and frequency during a typical week.

### 3.3. Physical Factors

Chronic conditions, including diabetes, dyslipidemia, or elevated blood glucose, malignancies, and liver disorders, were identified on the basis of self‐reported physician diagnoses. Participants were required to indicate whether a physician had diagnosed the disease (“yes” or “no”). The number of additional comorbidities was also recorded. Functional disability was evaluated by activities of daily living (ADLs), including dressing, transferring, eating, bathing, toileting, and continence. Participants who reported “difficulty but can still do it,” “need help,” or “cannot do it” for at least one ADL were considered to have an ADL disability.

### 3.4. Clinical Measures

Clinical assessments used were peak expiratory flow (PEF), body mass index (BMI), and waist circumference (cm). PEF (L/min) was measured using a peak flow meter (Shanghai, China). The participants were instructed to stand upright, breathe in deeply, place their lips tightly around the mouthpiece, and then breathe out rapidly and forcefully. The maneuver was performed three times, with a 30‐s interval between measurements [15]. The three measurements were recorded, and their average was used for the analysis. BMI (kg/m^2^) was determined as the body weight divided by the square of the height and used to group participants into the underweight (<18.5 kg/m^2^), normal (18.5–24.9 kg/m^2^), overweight (25.0–29.9 kg/m^2^), or obese (≥30.0 kg/m^2^) categories. Waist circumference was measured with a flexible measuring tape positioned over the clothing around the abdomen, level with the umbilicus [13]. Measurements were obtained at the end of expiration, with the umbilical level used as the anatomical reference point [16]. The waist circumference categories were <90/85 cm or ≥90/85 cm for men/women, respectively.

## 4. Statistical Analysis

### 4.1. Model Structure

Normally distributed continuous data are reported as means and standard deviations (SDs), while categorical variables are shown as frequencies and percentages. Skewed continuous data are described using medians and interquartile ranges (IQRs). Univariable and multivariable logistic regression were used to develop a model for predicting depression risk among patients with COPD. Each candidate predictor was initially examined using an unconditional univariable logistic regression model. Variables meeting the prespecified criterion for statistical significance were subsequently entered into the multivariable analysis. Stepwise variable selection in conjunction with the Akaike information criterion (AIC) was then applied to establish the final model specification. Regression coefficients, odds ratios (ORs), and 95% CIs were approximated using 1000 bootstrap replications to improve the stability and reduce potential bias in the parameter estimates [17]. Estimates were combined according to Rubin’s rules.

### 4.2. Internal Validation

The final multivariable model was validated using 1000 bootstrapping iterations. This procedure was used to correct for potential bias in the performance of the model.

### 4.3. Model Performance

The final predictive performance was evaluated according to its discrimination and calibration. Discrimination represents the ability of the model to differentiate between participants with depression risk and those who did not and was computed based on the area under the curve (AUC) of the receiver operating characteristic (ROC) curve. Calibration reflects consistency between the predicted and observed risk and was evaluated using the Hosmer‐Lemeshow test.

### 4.4. Clinical Scoring Tool

For practical application, a simplified points‐based clinical risk score was determined from the final prediction model. This approach is widely used to translate multivariable prediction models into tools that can be readily applied in clinical settings. The resulting score was designed to identify patients with COPD with a higher likelihood of developing depression within the subsequent 9 years. Continuous predictors were classified according to evidence from meta‐analyses and relevant guidelines for clinical practice. For categorical predictors, points were assigned by multiplication of the corresponding β coefficients (log odds) from the multivariable logistic regression model by two and rounding to the nearest whole number. The overall score was obtained by summing the points assigned to each predictor. Sensitivity, specificity, and AUC values were determined across various score thresholds, with the threshold yielding the maximum Youden index selected as the optimal cutoff [18]. The Youden index was determined as sensitivity + specificity − 1. The study was performed and reported per the Transparent Reporting of a Multivariable Prediction Model for Individual Prognosis or Diagnosis (TRIPOD) guidelines. All analyses were performed in SPSS 26. A two‐sided *p*  < 0.05 was deemed significant.

## 5. Results

### 5.1. Participants Information

Among the 851 participants included in the analysis, 434 developed depression risk during the subsequent 9 years. The cumulative incidence of depression over this time was 51.00%, with 62.87% of affected participants being male and 37.13% being female. The mean age was 62.96 years, and 535 participants (62.87%) were male. At baseline, 36.08% of participants reported difficulty with ADLs, and 32.90% had an abnormal waist circumference. Additional baseline information is presented in Table 1, together with information on the missing observations for all variables.

**Table 1:** Baseline characteristics of patients with COPD.

Variables	Total (*N* = 851)	No depression risk (*N* = 417)	Having depression risk (*N* = 434)	*p* ^a^
Age (years)	62.96 ± 10.30	67.24 ± 10.07	58.84 ± 8.74	**<0.001**
Male	535 (62.87)	271 (64.99)	264 (60.83)	0.228
Residence setting				0.058
Rural	749 (88.01)	358 (85.85)	391 (90.09)	—
Urban	102 (11.99)	59 (14.15)	43 (9.91)	—
Education level				**0.001**
Elementary school or below	54 (6.35)	30 (7.19)	24 (5.53)	—
Middle school	49 (5.76)	12 (2.88)	37 (8.53)	—
High school or above	748 (87.90)	375 (89.93)	373 (85.94)	—
Marital status				**<0.001**
Married/cohabiting	721 (84.72)	329 (78.90)	392 (90.32)	—
Others	130 (15.28)	88 (21.10)	42 (9.68)	—
Smoking status				**0.009**
Nonsmokers	358 (42.07)	159 (38.13)	199 (45.85)	—
Former smokers	152 (17.86)	90 (21.58)	62 (14.29)	—
Current smokers	341 (40.07)	168 (40.29)	173 (39.86)	—
Drinking status				0.358
Nondrinkers	552 (64.86)	278 (66.17)	274 (74.7)	—
Former drinkers	72 (8.46)	30 (7.19)	42 (14.4)	—
Current drinkers	227 (26.67)	109 (26.14)	118 (10.9)	—
Chronic conditions				0.243
1–2	455 (53.47)	214 (51.32)	241 (55.53)	—
≥3	396 (46.53)	203 (48.68)	193 (44.47)	—
Physical activity (MET)	274 (257–274)	274 (268–274)	274 (255–274)	**<0.001**
ADL difficulty	307 (36.08)	145 (34.77)	162 (37.33)	0.475
Cognitive function (score)	9.01 ± 3.45	9.00 ± 3.53	9.03 ± 3.38	0.899
BMI (kg/m^2^)				**0.002**
<18.5	98 (11.52)	62 (14.87)	36 (8.29)	—
18.5–23.9	493 (57.93)	247 (59.23)	246 (56.68)	—
24–27.9	178 (20.92)	70 (16.79)	108 (24.48)	—
≥28	82 (9.64)	38 (9.11)	44 (10.14)	—
Waist circumference (male/female) (cm)			—	0.145
<90/85	571 (67.10)	290 (69.54)	281 (64.75)	—
≥90/85	280 (32.90)	127 (30.46)	153 (35.25)	—
PEF (L/min)	163.6 (133.33–266.67)	163.60 (136.67–276.67)	163.60 (124.58–246.67)	**<0.001**

*Note:* Bold values indicate statistically significant inter‑group differences (*p* < 0.05) between COPD patients with and without depression risk were observed for age (*p* < 0.001), education level (*p*  =  0.001), marital status (*p* < 0.001), smoking status (*p*  =  0.009), physical activity (MET, *p* < 0.001), BMI category (*p*  =  0.002), and peak expiratory flow (PEF, *p* < 0.001). Patients with depression risk were younger and tended to have lower physical activity, poorer PEF, and a higher proportion of overweight or obesity.

Abbreviations: ADL, activities of daily living; BMI, body mass index; MET, metabolic equivalent; PEF, peak expiratory flow.

^a^Independent sample *t*‐tests, Fisher’s exact test, or chi‐squared tests were used to compare baseline characteristics with different depression risk.

### 5.2. Univariable and Multivariable Analyses

The logistic regression findings are provided in Table 2. The univariable analyses indicated that increasing age and non‐married status were inversely associated with the depression risk, whereas middle school education was significantly related to a higher risk. Compared with nonsmokers, former smokers had a greater likelihood of developing depression risk. Overweight status was also positively associated with depression risk, while higher PEF and greater physical activity were inversely associated with the outcome. In the multivariable analysis, six predictors were retained in the final prediction model: age, educational level, marital status, history of smoking, exercise, and PEF (Table 2). A model incorporating all candidate variables produced consistent findings (Supporting Information 1: Table S1). To further examine whether continuous predictors were related to depression risk in a nonlinear manner, restricted cubic spline (RCS) analyses were performed for age, PEF, and physical activity. The corresponding plots and tests for nonlinearity are provided in Supporting Information 2: Figures S1–S3.

**Table 2:** Results of logistic regression models for patients with COPD with depression risk.

Predictors	Total *N* (%)/mean ± SD	Having depression risk *n* (%)/mean ± SD	Univariable OR (95% CI)	Multivariable OR (95% CI)
Age (years)				
45–60	328 (38.54)	232 (53.46)	Reference	Reference
≥60	523 (61.46)	202 (46.54)	0.34 (0.26–0.46) ^∗∗^	0.34 (0.29–0.54) ^∗∗^
Sex				
Male	535 (62.87)	264 (60.83)	Reference	—
Female	316 (37.13)	170 (39.17)	1.20 (0.91–1.58)	—
Residence setting				
Rural	749 (88.01)	391 (90.09)	Reference	—
Urban	102 (11.99)	43 (9.91)	1.50 (0.99–2.28)	—
Education level				
High school or above	748 (87.90)	373 (85.94)	Reference	Reference
Middle school	49 (5.76)	37 (8.53)	3.10 (1.59–6.04) ^∗∗^	2.47 (1.31–4.65) ^∗∗^
Elementary school or below	54 (6.35)	24 (5.53)	0.80 (0.46–1.40)	1.05 (0.55–2.01)
Marital status				
Married/cohabiting	721 (84.72)	392 (90.32)	Reference	Reference
Others	130 (15.28)	42 (9.68)	0.40 (0.27–0.60) ^∗∗^	0.47 (0.29–0.78) ^∗∗^
Smoking status				
Nonsmokers	358 (42.07)	199 (45.85)	Reference	Reference
Former smokers	152 (17.86)	62 (14.29)	0.55 (0.38–0.81) ^∗∗^	0.46 (0.29–0.74) ^∗∗^
Current smokers	341(40.07)	173 (39.86)	0.82 (0.61–1.11)	0.76 (0.54–1.06)
Drinking status				
Nondrinkers	552 (64.86)	274 (74.7)	Reference	—
Former drinkers	72 (8.46)	42 (14.4)	1.42 (0.86–2.34)	—
Current drinkers	227 (26.67)	118 (10.9)	1.10 (0.81–1.50)	—
Chronic conditions				
1–2	455 (53.47)	241 (55.53)	Reference	—
≥3	396 (46.53)	193 (44.47)	0.84 (0.65–1.11)	—
Physical activity (MET)				
<250	575 (67.57)	56 (20.29)	Reference	Reference
≥250	276 (32.43)	220 (79.71)	0.63 (0.43–0.91) ^∗∗^	0.57 (0.38–0.86) ^∗∗^
ADL difficulty				
No	544 (63.92)	272 (62.67)	Reference	—
Yes	307 (36.08)	162 (37.33)	1.11 (0.84–1.48)	—
Cognitive function (score)				
<9	432 (50.76)	232 (53.46)	Reference	—
≥9	379 (44.54)	202 (46.54)	0.85 (0.65–1.11)	—
BMI (kg/m^2^)				
18.5–23.9	493 (57.93)	246 (56.68)	Reference	Reference
<18.5	98 (11.52)	36 (8.29)	0.58 (0.37–0.91) ^∗^	0.90 (0.52–1.54)
24–27.9	178 (20.92)	108 (24.48)	1.55 (1.09–2.20) ^∗^	1.34 (0.91–1.96)
≥28	82 (9.64)	44 (10.14)	1.16 (0.73–1.86)	0.99 (0.58–1.69)
Waist circumference (male/female) (cm)				
<90/85	571 (67.10)	281 (64.75)	Reference	—
≥90/85	280 (32.90)	153 (35.25)	1.24 (0.93–1.66)	—
PEF (L/min)				
<400	780 (91.66)	416 (95.85)	Reference	Reference
≥400	71 (8.34)	18 (4.15)	0.30 (0.17–0.52) ^∗∗^	0.26 (0.13–0.53) ^∗∗^

Abbreviations: ADL, activities of daily living; BMI, body mass index; MET, metabolic equivalent; PEF, peak expiratory flow.

^∗∗^ *p*  < 0.01.

^∗^ *p*  < 0.05.

### 5.3. Model Performance and Clinical Score Model

**Figure 1:**
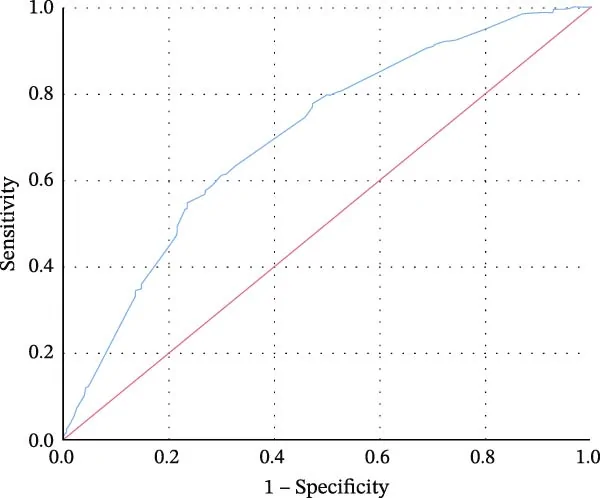
The discrimination curve for the model.

**Figure 2:**
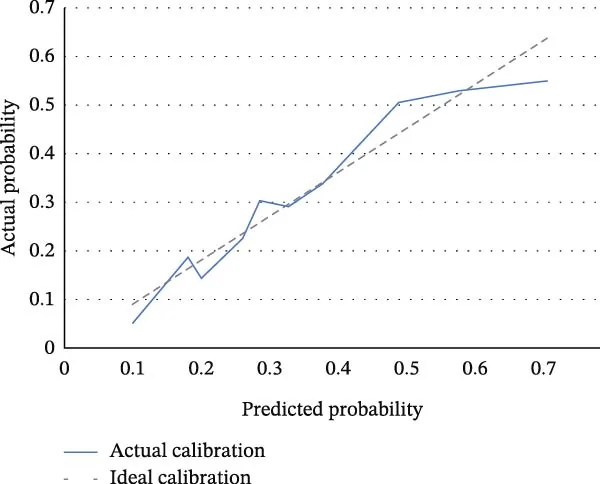
The calibration curve for the model.

The discrimination curve for the prediction model is presented in Figure 1. The final model showed satisfactory discrimination, with an AUC of 0.717 (95% CI: 0.683–0.751). The corresponding calibration curve is shown in Figure 2. The Hosmer‐Lemeshow test exhibited good agreement between predictions and observed risk (*χ* ^2^ = 9.400, *p* = 0.310).

A simplified clinical scoring system was subsequently constructed using the six predictors retained in the final multivariable model, as shown in Table 3. The total score ranged from 0, representing the lowest predicted risk, to 12, representing the highest predicted risk. This scoring system may facilitate the identification of patients with COPD at increased risk of depression within the following 9 years. The AUC of the clinical score was 0.717 (95% CI: 0.683–0.751). Based on the maximum Youden index (0.311), a score of ≥4 was identified as the optimal cutoff for predicting depression risk over the 9‐year follow‐up period, associated with a sensitivity of 61.2% and a specificity of 69.9%.

**Table 3:** Results of logistic regression models for COPD patients with depression risk.

Predictors	Risk score
Age (years)	
45–60	0
≥60	2
Education level	
High school or above	0
Middle school	2
Elementary school or below	0
Marital status	
Married/living together	0
Others	2
Smoking status	
Nonsmokers	0
Former smokers	2
Current smokers	0
Physical activity (MET)	
<250	2
≥250	0
PEF (L/min)	
<400	2
≥400	0

Abbreviations: MET, metabolic equivalent; PEF, peak expiratory flow.

## 6. Discussion

This study devised and internally validated a prediction model for estimating the 9‐year risk of depression among Chinese patients with COPD based on longitudinal CHARLS data. The final model was translated into a simple clinical scoring system designed to facilitate the identification of patients at elevated risk. The model incorporated six readily obtainable variables: age, educational level, marital status, BMI, exercise level, and PEF. This is the first predictive model that was developed specifically to estimate the 9‐year depression risk among patients with COPD in China. This model may provide a practical approach for identifying individuals who could benefit from early preventive strategies and closer psychological monitoring.

Notably, older age was inversely related to the depression risk in our study. COPD participants aged 60 years or older showed reduced likelihood of developing depression compared to those aged 45–60 years [19]. Several mechanisms may potentially explain this finding. Older adults may acquire more effective coping strategies and greater emotional resilience over the course of their lives, enabling them to adapt more successfully to the psychological burden of chronic disease. Previous studies have suggested that emotional regulation and psychological resilience may improve with advancing age, potentially providing some protection against depression [20]. In addition, selective survival may have contributed to this association. Older patients who remain represented in the study population may constitute a relatively healthier subgroup because individuals with severe depressive disorders may experience increased mortality and therefore be less likely to remain in longitudinal cohorts [21]. Nevertheless, our finding differs from previous evidence indicating that older individuals may be more susceptible to severe depression [19]. The relationship between age and depression risk in patients with COPD thus warrants further study.

Former smokers in this study had a higher risk of depression than never‐smokers. Tobacco smoke contains numerous toxic gases and particulate constituents that can contribute to persistent airway inflammation and airflow obstruction, ultimately impairing pulmonary function [22]. The relationship between smoking cessation and depression risk may also reflect reverse causation or an underlying health status. In our analysis, former smokers, rather than current smokers, showed an increased risk relative to those who had never smoked. One possible explanation is that patients may have stopped smoking because of worsening physical health or the progression of COPD, and the resulting decline in physical functioning may itself adversely affect psychological well‐being [23].

Lower educational attainment was positively associated with depression risk among patients with COPD, consistent with previous evidence showing that lower education is associated with depressive symptoms [24]. Educational attainment may influence depression through several interrelated pathways. Individuals with less education may have lower health literacy, which can make it more difficult to understand COPD self‐management recommendations, adhere to prescribed therapies, and recognize early deterioration in symptoms. Inadequate disease management and poorer disease control may subsequently contribute to psychological distress [24]. Lower educational attainment may also be associated with financial difficulties, reduced access to healthcare, unemployment, unstable housing, and other persistent stressors, all of which may increase vulnerability to depression [24].

Marital status was another factor that was positively associated with the depression risk. Spouses may experience substantial caregiving demands and emotional burden when supporting a partner with chronic respiratory disease, potentially increasing stress within the household [25]. Conversely, widowhood or separation may eliminate some of these caregiving‐related pressures, particularly when the previous relationship was characterized by substantial conflict or when caregiving demands had become unsustainable. Individuals who are widowed or otherwise no longer partnered may also establish alternative sources of social support, including relationships with adult children, relatives, or community organizations, which may better address their specific needs.

Physical activity was inversely associated with depression risk and represents an important modifiable factor in our prediction model. This finding is consistent with evidence that physical activity can provide meaningful mental health benefits even when performed at levels below current public health recommendations [26]. Dina et al. reported an association between physical activity and depression across 13 studies, with the relationship remaining consistent regardless of the method used to measure physical activity. Exercise is particularly relevant to patients with COPD because structured physical activity and exercise training can improve exercise tolerance, enhance skeletal and respiratory muscle function, reduce dyspnea and fatigue, and enhance quality of life [27].

Of the clinical measures evaluated herein, PEF was identified as an important predictor of depression risk. Participants with higher PEF values were less likely to develop depression risk. PEF provides an accessible measure of expiratory airflow and therefore reflects aspects of pulmonary function. Previous research has demonstrated associations between impaired lung function and adverse health‐related outcomes [23], while Hu et al. [28] similarly reported an association between lung function and depression. These findings further support the importance of maintaining pulmonary function and may provide additional justification for physician counseling regarding pulmonary rehabilitation and other interventions aimed at preserving physical capacity in patients with COPD.

We subsequently converted the final multivariable model into a simple clinical scoring system to facilitate the estimation of 9‐year depression risk. The score incorporates six routinely available clinical characteristics, allowing for risk assessment without specialized laboratory testing or complex calculations. The tool could potentially be used by clinicians to identify patients who warrant closer psychological assessment and preventive intervention and, with appropriate validation, may also support patient self‐awareness regarding long‐term depression risk. Categorizing continuous variables improves the practicality of a point‐based scoring system but inevitably results in some loss of statistical information. The spline analyses presented in the Supporting Information Figure illustrate the relationships between key continuous predictors and the log‐odds of depression risk and provide additional information regarding their functional forms. Although the model demonstrated acceptable predictive performance, further refinement may be necessary before application to populations with characteristics different from those of the derivation cohort. Incorporating additional clinical variables or biomarkers may also improve risk discrimination and provide greater insight into the biological pathways linking COPD with depression.

### 6.1. Limitations

Several limitations must be noted. First, because CHARLS enrolls adults aged ≥45 years, the applicability of our findings to younger patients with COPD is uncertain, and the model should therefore be interpreted primarily in the context of this older population. Second, the CHARLS database does not provide sufficient information to stratify participants according to COPD severity, preventing a detailed assessment. We attempted to partially address this limitation by incorporating PEF as an objective measure of pulmonary function and a proxy for disease severity. Third, the prediction model was developed using a nationally representative Chinese cohort but has not yet undergone external validation. Finally, COPD status in CHARLS was determined on the basis of self‐reported physician diagnosis rather than spirometric confirmation. Although this approach was necessary for the analysis of this large national longitudinal cohort, reliance on self‐reported diagnosis may result in some degree of disease misclassification.

## 7. Conclusions

Here, we designed and internally validated the first model specifically designed to estimate the 9‐year risk of depression among patients with COPD in China. The accompanying point‐based scoring system uses six readily available variables and may facilitate the early identification of individuals at particularly high risk in clinical and community settings. Earlier recognition of elevated risk could support more timely patient education, psychological assessment, and targeted modification of relevant risk factors, potentially contributing to a reduction in the burden of depression among individuals with COPD.

## Author Contributions

Cui Wang, Mengya Wang, Nan Zhang, and Yanying Bi conceptualized the study. Cui Wang collected data and wrote the manuscript. Cui Wang, Mengya Wang, and Nan Zhang analyzed data.

## Funding

This study was supported by the Research Project of Zhejiang Chinese Medical University (Grant 2024RCZXZK36) and the Undergraduate Innovation and Entrepreneurship Training Program Project (Grant 202610344048).

## Disclosure

All authors read and approved the final manuscript.

## Ethics Statement

All participants signed informed consent. The survey was approved by the Institutional Review Board (IRB) at Peking University (IRB Approval Number IRB00001052‐11015).

## Consent

The authors have nothing to report.

## Conflicts of Interest

The authors declare no conflicts of interest.

## Supporting Information

Additional supporting information can be found online in the Supporting Information section.

## Supporting information


**Supporting Information 1** Table S1: The results of the fully adjusted multivariable logistic regression model incorporating all candidate variables for depression risk, which verifies the robustness of the final parsimonious prediction model presented in Table S2.

**Supporting Information 2** Figures S1–S3: The results of RCS analyses, which were conducted to evaluate potential nonlinear relationships between continuous predictors (age, PEF, and physical activity) and depression risk. The formal nonlinearity tests further supported the existence of significant nonlinear trends for all three indicators.

## Data Availability

The data that support the findings of this study are available upon request from the corresponding author. The data are not publicly available due to privacy or ethical restrictions.
