# Supplementary material for: A Prediction Model for 9‐Year Depression Risk Among Patients With COPD in China
Source: Depress Anxiety. 2026 Sep 18;2026:5536644. doi: 10.1155/da/5536644 (PMC13586986; doi:10.1155/da/5536644)
Supplement: Supplementary file 1 [file DA-2026-5536644-s001.docx]

****Supplementary Figure 1.** Restricted cubic spline plots of the associations between age and depression risk**

***P* for nonlinearity = 0.0418**

****Supplementary Figure 2.** Restricted cubic spline plots of the associations between PEF and depression risk**

***P* for nonlinearity = 0.0168**

****Supplementary Figure 3.** Restricted cubic spline plots of the associations between physical activity and depression risk**

***P* for nonlinearity = 0.0214**
