# Supplementary material for: A Prediction Model for 9‐Year Depression Risk Among Patients With COPD in China
Source: Depress Anxiety. 2026 Sep 18;2026:5536644. doi: 10.1155/da/5536644 (PMC13586986; doi:10.1155/da/5536644)
Supplement: Supplementary file 2 [file DA-2026-5536644-s002.docx]

**Supplementary Table 1** Unadjusted and fully adjusted models for patients with COPD with depression risk

| Predictors | Total  N(%)/Mean±SD | Having depression risk  n(%)/Mean±SD | Univariable OR  (95% CI) | Multivariable OR  (95% CI) |
| --- | --- | --- | --- | --- |
| Age, years |  |  |  |  |
| 45～60 | 328(38.54) | 232(53.46) | Reference | Reference |
| ≥60 | 523(61.46) | 202(46.54) | 0.34 (0.26–0.46)** | 0.42 (0.29–0.62)** |
| Sex |  |  |  |  |
| Male | 535(62.87) | 264(60.83) | Reference | Reference |
| Female | 316(37.13) | 170(39.17) | 1.20 (0.91–1.58) | 1.20 (0.72–1.94)** |
| Residence setting |  |  |  |  |
| Rural | 749(88.01) | 391(90.09) | Reference | Reference |
| Urban | 102(11.99) | 43(9.91) | 1.50 (0.99–2.28) | 0.62 (0.38–1.01) |
| Education level |  |  |  |  |
| High school or above | 748(87.90) | 373(85.94) | Reference | Reference |
| Middle school | 49(5.76) | 37(8.53) | 3.10(1.59–6.04)** | 3.22 (1.42–7.31)** |
| Elementary school or below | 54(6.35) | 24(5.53) | 0.80 (0.46–1.40) | 1.31 (0.58–2.94) |
| Marital status |  |  |  |  |
| Married/cohabiting | 721(84.72) | 392(90.32) | Reference | Reference |
| Others | 130(15.28) | 42(9.68) | 0.40 (0.27–0.60)** | 0.43 (0.24–0.76)** |
| Smoking status |  |  |  |  |
| Non-smokers | 358(42.07) | 199(45.85) | Reference | Reference |
| Former smokers | 152(17.86) | 62(14.29) | 0.55 (0.38–0.81)** | 0.52 (0.28–0.96)* |
| Current smokers | 341(40.07) | 173(39.86) | 0.82 (0.61–1.11) | 0.78 (0.47–1.29) |
| Drinking status |  |  |  |  |
| Non-drinkers | 552(64.86) | 274(74.7) | Reference | Reference |
| Former drinkers | 72(8.46) | 42(14.4) | 1.42 (0.86–2.34) | 0.96 (0.45–2.03) |
| Current drinkers | 227(26.67) | 118(10.9) | 1.10 (0.81–1.50) | 1.00 (0.63–1.59) |
| Chronic conditions |  |  |  |  |
| 1-2 | 455(53.47) | 241(55.53) | Reference | Reference |
| ≥3 | 396(46.53) | 193(44.47) | 0.84 (0.65–1.11) | 0.94 (0.65–1.35) |
| Physical activity, MET |  |  |  |  |
| <250 | 575(67.57) | 56(20.29) | Reference | Reference |
| ≥250 | 276(32.43) | 220(79.71) | 0.63 (0.43–0.91)** | 0.48 (0.26–0.91)* |
| ADL difficulty |  |  |  |  |
| No | 544(63.92) | 272(62.67) | Reference | Reference |
| Yes | 307(36.08) | 162(37.33) | 1.11 (0.84–1.48) | 0.69 (0.44–1.10) |
| Cognitive function, score |  |  |  |  |
| <9 | 432(50.76) | 232(53.46) | Reference | Reference |
| ≥9 | 379(44.54) | 202(46.54) | 0.85(0.65–1.11) | 1.25(0.86–1.82) |
| BMI, kg/m^2^ |  |  |  |  |
| 18.5~23.9 | 493(57.93) | 246(56.68) | Reference | Reference |
| <18.5 | 98(11.52) | 36(8.29) | 0.58 (0.37–0.91)* | 1.38 (0.71–2.71) |
| 24~27.9 | 178(20.92) | 108(24.48) | 1.55 (1.09–2.20)* | 1.75 (0.84–3.64) |
| ≥28 | 82(9.64) | 44(10.14) | 1.16 (0.73–1.86) | 1.67 (0.72–3.89) |
| Waist circumference  （male/female）, cm |  |  |  |  |
| <90/85 | 571(67.10) | 281(64.75) | Reference | Reference |
| ≥90/85 | 280(32.90) | 153(35.25) | 1.24 (0.93–1.66) | 1.05 (0.61–1.82) |
| PEF, L/min |  |  |  |  |
| <400 | 780(91.66) | 416(95.85) | Reference | Reference |
| ≥400 | 71(8.34) | 18(4.15) | 0.30 (0.17–0.52)** | 0.34 (0.15–0.76)** |

ADL: activities of daily living; MET: metabolic equivalent; BMI: body mass index; PEF: peak expiratory flow.

**: *P*<0.01; *: *P*<0.05.
